# Progress in Medicine

**Published:** 1900-02-10

**Authors:** 


### Progress in Medicine.

### FEVERS.

(*Continued from page* 292.)

**Typhoid in India.**—The losses of the English army from typhoid are actually increasing, in spite of endless care and discussions. Over a third of all the deaths in 1897 were due to it. These 638 men represent about nine per 1,000 of the total forces. The majority of attacks are in the younger men, and the mortality of those attacked is much higher than is the rule in this country, so that instead of a rate of from 9 to 16 per cent., we find the Indian mortality among soldiers rises to 25 per cent. of all cases of the disease.[16] Every precaution and investigation have been so far failures. There are theories that it is water-borne, carried by flies, dust, native cooks, due to over-eating, drinking, or to the youth of the soldiers, or the proximity of the bazaars. The Government orders this or that means of prevention, but goes on losing every year half a battalion by death, while two more full battalions are useless invalids from this one disease. No other disease comes near its fatal proportions; even dysentery shows little more than a quarter of the deaths caused by enteric. In France, a military epidemic at Cherbourg led to an investigation by Mollet,[17] who condemned the water supply, but also found the bacillus in the dust of the woodwork of the dormitories where the men both eat and sleep. He condemns, too, the Chamberland filter as too liable to get out of order, unless it is tested daily by the medical officer. The British Government, failing in all its endeavours to stop the conveyance of the infection, has permitted the trial of Professor A. E. Wright's anti-typhoid vaccine on a large scale, both in Africa and India. The results in the few cases where it was used in the Soudan are encouraging, and the prevalence of the scourge in Ladysmith, Mafeking, and elsewhere make us hope it will prove an effective safeguard. A pamphlet giving Professor A. E. Wright's own account of it shows that animals inoculated with sterilised cultures of typhoid bacilli obtain a great power of resistance to infection from living ones, and in men these injections produce the same blood changes as they do in animals, which changes are identical with those caused by an actual attack of the disease. Thus, the phenomena of agglutination and sedimentation are found in those who are inoculated just as in convalescents from typhoid. These effects last in many cases from one to two years, but time alone will show the true duration of the protection. Wright also points out that the inoculations will not cure an attack of fever already present, and indeed the immunity does not commence till several days after the injections have been given. He recommends a second inoculation a week or more after the first, as it increases the protection to be derived from it. The Bill for regulating the oyster fisheries to prevent the communication of typhoid has been changed by the House of Lords Committee, who have substituted the Sea Fisheries' Committees as the administrative bodies, instead of the county councils. The *Lancet*[18] holds that this will wreck the measure, and urges that responsible sanitary authorities should have power to look after the growth not only of oysters but also of other shell fish.

A remarkable case of the failure of the Widal reaction from some idiosyncrasy of the patient is reported by H. Batty Shaw.[19] The patient had the ordinary symptoms of typhoid, including spots and an enlarged spleen, but on the fourth and fifth days of the illness the Widal reaction was negative. An abscess formed in the abdominal wall, and from this the typhoid bacillus was obtained. The patient's blood failed to cause clumping in the bacilli from this source, though he was then in the second month of his illness. A remarkable theory of the causation of relapse was given by J. C. Wilson[20]

at the annual meeting of the Ontario Medical Association. He holds that when the increased movements of the patient and the consumption of more solid food produce more energetic peristalsis, the contents of the gall-bladder (which often contain a pure culture of the bacillus) are driven out into the intestines, and produce a reinfection. Thus there is a fresh infection before immunity is complete. It has been objected, however, that this theory hardly explains why we have relapses after 30 days or later. At the Aberdeen Asylum a case has been reported where the Widal reaction was positive, but the result of the post-mortem showed the absence of typhoidal changes and the presence of ulcerative endocarditis.

**Tetanus.**—Carless[21] gives a summary of the results of intracerebral injection of antitoxin. In 25 recorded cases he finds 11 recoveries and 14 deaths, which he considers satisfactory, since several of the recoveries were in severe cases, *i.e.*, in persons where the symptoms were early in appearing or rapid in their evolution. He expects a better percentage of recoveries will be shown when we are able to distinguish those cases where the bulb centres are already implicated and recognise them as hopeless for treatment. He mentions one case where the injections into the brain were made five times, and 71 cc. of serum were employed, besides other hypodermic ones. Recovery from tetanus took place, but in a later communication Gibb has since announced the death of the patient from pus formation eight weeks after the last injection. It is needless to say that extreme care had been taken to avoid sepsis. Leyden[22] reports a puerperal case where the incubation period lasted 10 days, and the symptoms were severe. Injections were twice made into the brain, besides others subcutaneously, with slowly progressive results. By the seventeenth day of the attack nearly all symptoms had disappeared. Leyden reckons the mortality of cases with an incubation period of one to eight days at 91 per cent., and of those over 14 days at 52 per cent. The carbolic acid treatment, known as that of Bacelli, is an important rival to the antitoxin method, and has been highly recommended by H. C. Wood and Babes. The latter injects a half per cent. solution of carbolic acid along the spine from the neck downward in doses of 5 to 10 grammes every two hours. This is increased day by day very rapidly. Ascoli injects a 2 or 3 per cent. solution some 10 times in the 24 hours, containing in all ·35 of a gramme of carbolic, or about 5 grains. Flavel Woods[23] reports a successful case, and is in favour of heroic doses. Henderson, in India, used two drops of carbolic in 20 of water three times a day, and reports seven recoveries out of 20 cases. H. C. Wood[24] quotes from Ascoli the following table of the results of the different treatments:—

| Method. | Cases. | Deaths. | Mortality. |
|---|---|---|---|
| Bacelli ... | 34 ... | 1 ... | 3·2 |
| Tizzoni ... | 42 ... | 7 ... | 17·8 |
| Behring ... | 28 ... | 10 ... | 35·7 |

He adds that it is remarkable what doses of carbolic are well borne by tetanus patients, so that nine grains have been injected daily with no symptoms of an overdose. Bacelli commences with daily doses of three grains in a two per cent. dilution, keeps the patient very quiet and carefully disinfects any wound which exists. These statistics, so far as they go, certainly show that the treatment has considerable value, and is well worthy of further investigation. The acid appears to act directly on the poison, and also as a nerve sedative.

[16] Lancet, Aug. 19; and Indian Med. Rec., June 7. [17] Lancet, Aug. 5. [18] Lancet, Oct. 7. [19] Brit. Med. Jour., Nov. 4. [20] Med. Rec., July 8. [21] Practitioner, July. [22] Brit. Med. Jour., Aug. 5. [23] Med. Rec., Oct. 4. [24] Brit. Physician, Sept.; and Merck's Archives, May.

### DISEASES OF THE RESPIRATORY SYSTEM.

**Pneumonectomy.**—The operation of pneumonectomy, that is, the excision of portions of diseased lung, is advocated by Pyle,[1] who has made a series of successful experiments on dogs. He regards it as peculiarly suitable in cases of apical phthisis. The pleural cavity having been opened, the diseased portion of lung is dragged into the wound, so as to prevent the ingress of air, ligatured with silkworm gut, excised, and the stump treated with the actual cautery. The great danger of the operation is from hæmorrhage. Matas[2] mentions that this operation was successfully performed by Fuffier in 1894 upon a human being. The chief difficulty according to this author is the prevention of acute traumatic pneumothorax, which may result in sudden death from asphyxia, or from reflex cardio-respiratory paralysis. To guard against this operators have attempted first to form adhesions between the chest wall and visceral pleura, either by means of irritants or caustics, by prolonged acupuncture, or by actually suturing the surfaces together. None of these methods are, however, so satisfactory as that proposed and carried out by Fell, who introduces into the windpipe a cannula connected with bellows, and by this means keeps up a pressure within the lung sufficient to prevent its collapse. Great diversity of opinion exists as to the best method of treating hæmorrhage from a wound penetrating the lung, some authorities claiming that it is best to immediately seal or plug the wound and strap the side, others that it is better by inserting a tube into the pleural cavity to allow the lung to collapse, and thus allay the bleeding. A discussion on this point will be found in the "Annals of Surgery," June, 1899, and a series of cases illustrating the point in question in the New York *Medical Journal*, August 26th, 1899. Delorme[3] thinks it advisable that in very severe hæmorrhage the lung should be exposed and the bleeding point secured, the risk of pneumothorax being disregarded.

**Pleural Effusions.**—With regard to the physical signs of fluid in the pleura, Bishop[4] points out that flatness on percussion is the most constant, and is accompanied by a sense of resistance to the percussing finger; the percussion note, however, may sometimes be tympanitic, and in the same way bronchial breathing is as common as absence of breath-sounds. Indeed, the physical signs of pleural effusion are not infrequently contradictory. Thus Schüle[5] reports two cases of pleurisy with effusion in which the friction-rub of dry pleurisy was present, due to the fact that circumscribed areas of lung and costal pleura were in contact, the exudate not being very great. A series of cases is also reported by Stick,[6] showing how easily purulent accumulations in children may be overlooked, and how in the presence of adhesions even puncture may be unreliable, as purulent and serous pleurisy may be present on the same side at the same time. A case of empyema sacculated between the heart and the lung is reported by Dodd.[7] The patient, a man of 57 years, had an attack of pneumonia on February 20th, and this was followed by an empyema at the base of the left lung, which was opened and drained. The temperature still kept high, and death ensued on March 13th. At the post-mortem an encapsuled abscess was found between the heart and lung. The abscess was in relation to the anterior wall of the chest, in the fourth intercostal space from the mammary line to about 3 cm. towards the sternum. During life there had been dulness from the third rib downwards, and from the edge of the sternum to the mammary line. There had also been a tender spot at the junction of the fifth rib with the sternum, and the bone at this point was found necrosed. Writing of the diagnosis between supraphrenic and subphrenic pyo-pneumothorax, Short[8] gives the following points of distinction: In supraphrenic abscess there is generally a history of antecedent lung disease, sudden pain in the affected side, coughing, and dyspnœa. If the lesion be on the right side the liver is pushed down, the heart pushed to the left, with no resonant area between the dulness due to these organs. If the mischief on the left side be above the diaphragm, then the heart will be pushed to the right. In supraphrenic cases the diaphragm is pushed down and is motionless. On the other hand, subphrenic abscess is generally a sequel to digestive disorders, and if consequent upon rupture of a gastric ulcer is accompanied by sudden pain in the epigastrium, tenderness, and vomiting. The liver in such cases is generally depressed, the heart raised, but the diaphragm moves freely. Purulent effusions situated immediately above or below the diaphragm may perforate and connect the pleural and peritoneal cavities, but it is very rarely that simple effusions do so. Such a case is, however, reported by James.[9] The patient, a labourer of 48 years of age, was admitted into the Edinburgh Royal Infirmary suffering from ascites and fluid in the right pleura. It was found that by tapping either the abdomen or chest the fluid from both cavities could be evacuated, and this operation was repeated ten times, the average quantity of fluid being 245 ounces. The fluid was greenish, milky, and its specific gravity varied from 1,006-10. No cause for the condition could be assigned, nor could the position of the opening between the right pleura and peritoneum be located. A condition of the pleura not generally recognised is described by Hodenpyl,[10] namely, miliary tuberculosis without other tuberculous involvement of the lung. In this condition small miliary tubercles are found scattered over the visceral pleura; these are prone to become fibrous, but in susceptible persons may assume unusual importance by setting up a generalised tuberculous exudative pleurisy, or by complicating through concurrent infection an exudative pleurisy of independent origin. Another condition of some rarity is non-suppurative peripleuritis,[11] of which Lavallée records two cases. With this condition there is intense thoracic pain and auscultatory signs of pleurisy, but the inflammatory process is in the cellular tissue beneath the pleura. The disease may terminate in suppuration. Treatment is similar to that of pleurisy.

**Pneumonia.**—Whitla[12] regards lobar pneumonia as an acute specific fever, of which the lung condition is merely a local lesion. This is evidenced by the fact that the fever may exist for days before lung symptoms appear, and may subside although evidence of consolidation still exists. Indeed, the lung may never be attacked at all, the only lesion being acute pleurisy or empyema. The organism most commonly associated with the disease is Fränkel's pneumococcus; less common are Friedländer's bacillus, streptococcus, staphylococcus, aureus, and the organisms of tuberculosis, influenza, and enteric, or Klein's bacillus of the Middlesborough epidemic. Acute tuberculo-pneumonic phthisis he has seen on one occasion only, and the association of pneumonia and erysipelas he regards as due to their common origin from the streptococcus. Influenzal pneumonia may be streptococcic, diplococcic, or mixed. It is a clinical entity distinguished by the absence of pleural pain and nasty sputum, by its patchy distribution, and by its progressive spread. Dulness of percussion note is seldom well marked, and the duration of the disease is much prolonged. Influenzal pneumonia is contagious. Secondary pneumonia as seen in Bright's disease, typhoid, measles, and diabetes, are identical in pathology and bacteriology with these already referred to. Palier[13] describes the following varieties of atypical pneumonia: (1) Gastric, accompanied by abdominal cramps, tenderness, and constipation, due, probably, to vagus disturbance; (2) cerebral, in which meningitis ensues; (3) wandering pneumonia, a second lobe or second lung being attacked as soon as the primary lesion has quieted; (4) abortive pneumonia, in which, with high temperature, rapid respiration, and other symptoms of a few days' duration, the physical signs do not plainly manifest themselves; (5) chronic, croupous, and catarrhal cases, subacute from the onset, lasting for months, and frequently terminating in complete recovery. These resemble cases of phthisis, but clear up, leaving no trace behind. Under this head are included cases of prolonged resolution; (6) masked pneumonia, in which physical signs do not appear till the crisis, and then even imperfectly. Palier thinks that a good prognosis may always be given in the case of children if not more than two lobes of the same lung are involved.

1 New York Med. Jour., June 10, 1899. 2 Annals of Surgery, June, 1899. 3 La Semaine Médicale, June 14, 1899. 4 Med. Rec., July 29, 1899. 5 Münch Med. Woch, No. 51, 1898. 6 Pa. Med. Jour., July, 1898. 7 Med. Rec., Oct. 7, 1899. 8 Birm. Med. Rec., April, 1899. 9 Brit. Med. Jour., July 29, 1899. 10 Med. Rec., June 24, 1899. 11 Rev. Thérap. Med. Chi., May 1, 1899. 12 Dub. Jour. Med. Sci., April, 1899. 13 New York Med. Jour., Sept. 16, 1899.

